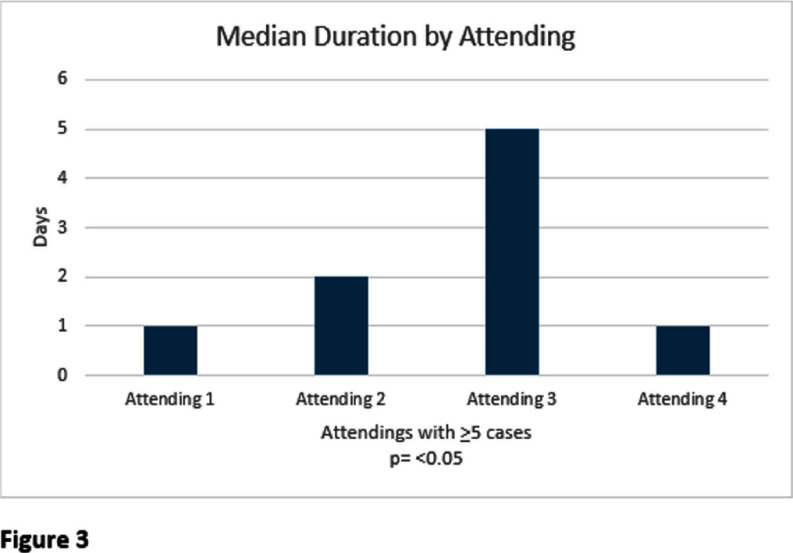# Evaluation for Urologic Surgery Antimicrobial Prophylaxis Practices at a Large Academic Medical Center

**DOI:** 10.1017/ash.2025.250

**Published:** 2025-09-24

**Authors:** Michael Vala, Christopher McCoy, Ryan Chapin

**Affiliations:** 1 Beth Israel Deaconess Medical Center; 2 Beth Israel Lahey Health System; 3 Beth Israel Deaconess Medical Center

## Abstract

**Background:** The 2019 American Urological Association (AUA) best practice statement for Urologic Procedures and Antimicrobial Prophylaxis recommends single-dose peri-operative antimicrobial prophylaxis (e.g. cefazolin or trimethoprim/sulfamethoxazole) not to continue past closure of the incision for Class I and II genitourinary (GU) procedures even in the presence of asymptomatic bacteriuria (ASB). Class I and II GU procedures encompass the majority of urologic procedures which are clean procedures in low risk patients and clean-contaminated procedures, respectively. The objective of this study is to assess current urologic antimicrobial prophylaxis practices at a large academic medical center. **Methods:** This retrospective observational study included adults who underwent a urologic procedure from September-October 2024 to assess AUA guideline adherence. Patients with a history of renal transplant, documented concern for symptoms consistent with urinary tract infection prior to the procedure, or receiving antibiotics for another condition were excluded. Both inpatient and outpatient preprocedural, intra-operative and postprocedural antibiotics were evaluated. Pre-procedural urine cultures results, attending of record, and type of procedure were correlated with prophylaxis practices using a one way ANOVA. **Results:** Of the 80 patients reviewed 41.3% received only single dose pre-operative prophylaxis and 57.5% received Discussion:

Nearly half of patients who underwent urologic procedures had a prophylaxis duration of < 2 4 hours in concordance with the AUA best practice recommendations. Opportunities exist for optimizing agent selection education. No difference in length of prophylaxis was found to correlate between different procedures performed. The presence of pre-operative ASB and ordering attending were found to correlate with an increased duration of prophylaxis. A future institutional practice guideline and order set for urologic procedure antimicrobial prophylaxis may be necessary to optimize agent selection and duration for these GU procedures.

**Figure:**
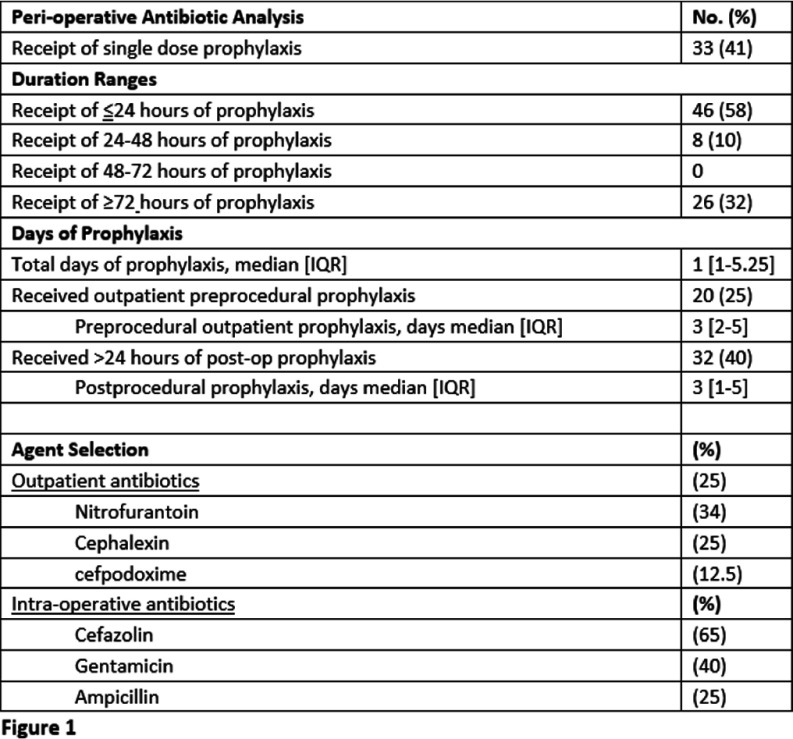

**Figure:**
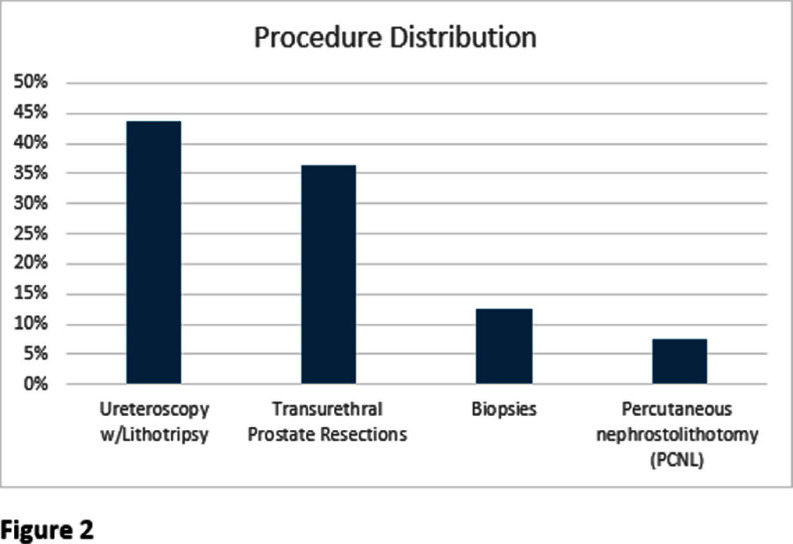

**Figure:**